# Projecting Climate-Induced Shifts in the Richness and Spatial Distribution of Invasive Alien Plants Across China Under Alternative Shared Socioeconomic Pathways

**DOI:** 10.3390/plants15111680

**Published:** 2026-05-29

**Authors:** Wen Lu, Mao Lin, Siyu Liu, Bao Liu

**Affiliations:** 1 School of Civil Engineering and Architecture, Southwest University of Science and Technology, Mianyang 621002, China; leuven@swust.edu.cn; 2 Forestry and Landscape Gardening Bureau, Qingshen County, Meishan 620400, China; 13408543373@163.com; 3 College of Forestry, Fujian Agriculture and Forestry University, Fuzhou 350002, China; 13881976640@163.com

**Keywords:** climate change, MaxEnt, richness distribution pattern, biological invasion, invasive plants

## Abstract

Climate change is profoundly altering species’ geographical distributions, with particularly pronounced effects on the richness patterns of invasive alien plants. As China represents a global hotspot for biological invasions, accurately projecting these shifts is imperative for formulating proactive and effective management strategies. This study integrated occurrence records for 321 invasive plant species with seven key environmental predictors within a MaxEnt modeling framework, supplemented by ArcGIS v10.8 spatial analysis, to simulate potential species richness distributions under current climatic conditions and three future periods (2050s, 2070s, and 2090s) across three Shared Socioeconomic Pathways (SSP126, SSP245, and SSP585). The optimized models exhibited strong predictive performance (mean AUC = 0.972 ± 0.037; mean TSS = 0.877 ± 0.115), with 92.1% of species achieving AUC > 0.9. Annual precipitation metrics emerged as the predominant drivers, with precipitation of the driest month (Bio14, 37.6%), annual precipitation (Bio12, 15.6%), and minimum temperature of the coldest month (Bio6, 13.8%) exerting the strongest influence on species distributions. Contemporary invasive plant richness hotspots are concentrated in southern and southwestern China. Under future climate scenarios, substantial range shifts are anticipated: suitable habitats are projected to expand significantly for 58 species (a mean change of +145.8%), while contracting for 24 species (a mean change of −50.4%). Notably, the centroid of maximum species richness is projected to undergo a pronounced north-northwestward displacement, migrating from its current location in Xiangcheng District, Hubei Province, to Lushi County, Henan Province, by the 2090s under the SSP585 scenario. This trajectory coincides with a marked expansion of areas characterized by medium and high species richness, a trend that is particularly accentuated under the high-emission pathway. In conclusion, this study provides a robust, spatially explicit assessment of the future dynamics of invasive plant richness in China, highlighting a significant north-northwestward redistribution under climate change. These findings establish a critical scientific baseline for prioritizing regional monitoring efforts and implementing preemptive control measures in areas facing heightened invasion risk.

## 1. Introduction

Anthropogenic global warming is inducing persistent and long-term shifts in the geographical distributions of both native tree species and invasive alien taxa [1,2], thereby fundamentally restructuring ecosystem form and function, cascading into impacts on ecosystem services and human well-being, and feeding back into the dynamics of the climate system [3]. Biological invasions inflict substantial and escalating environmental, social, and economic damage worldwide [4], elevating the management of invasive alien species (IAS) to one of the most urgent global environmental imperatives of the 21st century [5]. Although numerous international and national initiatives have been launched to curb biodiversity loss since the mid-20th century [6], persistent conservation gaps remain, with analyses suggesting that an additional 35.3% of global land area may require some form of protection to safeguard biodiversity effectively [7]. Against this backdrop, accurately forecasting climate-driven changes in the richness and spatial dynamics of invasive plant assemblages is indispensable for devising preemptive and effective management interventions [8].

China stands out as a global epicenter for biological invasions [9]. Propelled by rapid economic development and international trade, the number of alien plant species established in China has surged to over 660 by 2020, posing severe threats to native biodiversity, ecosystem services, and agricultural productivity [10]. The cumulative economic toll attributed to just eleven major invasive species in China since 1980 is estimated at a staggering 2363.5 billion US dollars [11]. Climate change projections consistently forecast an exacerbation of invasion pressures [12]. While prior investigations have explored the potential range expansions of individual or small groups of invasive plants in response to rising temperatures across China, studies that comprehensively address the large-scale patterns of multi-species richness and community-level dynamics under future climate scenarios are notably limited.

Ecological Niche Models (ENMs), exemplified by Species Distribution Models (SDMs), constitute a cornerstone methodology for projecting species’ biogeographic responses to climate forcing [13]. These models elucidate statistical relationships between known species occurrences and environmental predictors to estimate spatial patterns of habitat suitability [14]. The extant literature contains numerous SDM applications for projecting the distributions of single or a limited number of invasive plant species in China [15,16,17,18]. Nevertheless, comprehensive, multi-species syntheses that integrate richness mapping with spatially explicit analyses of centroid shifts and compositional turnover under a suite of future climate scenarios remain limited [19,20].

**Figure 1:**
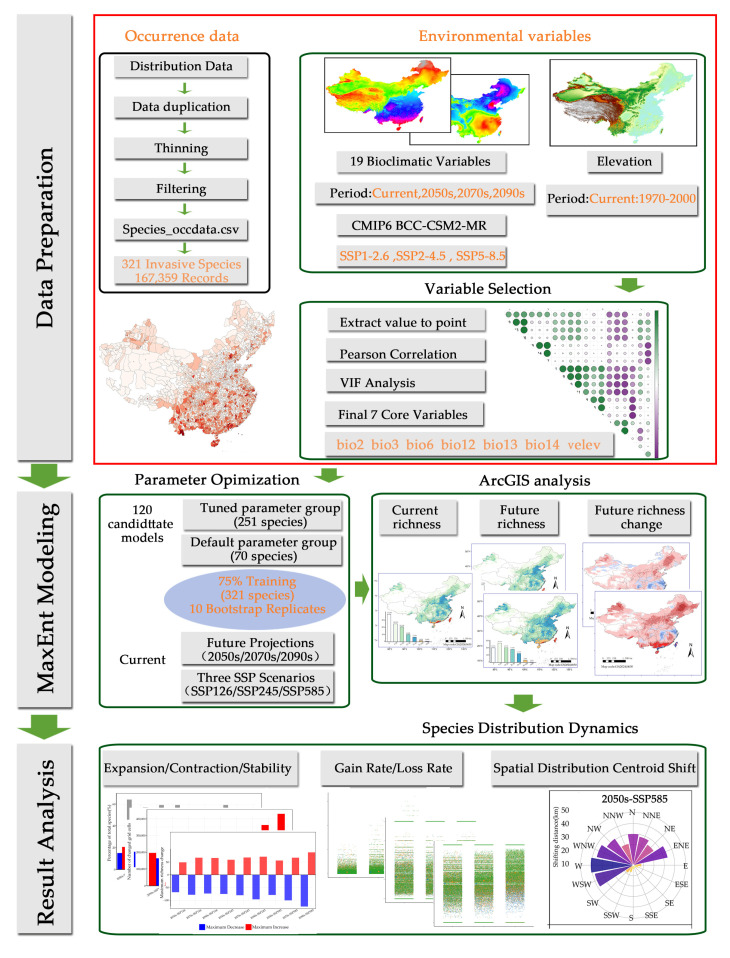
Analytical workflow of the study on invasive plant richness distribution under climate change.

To address this critical knowledge gap, the study employs an optimized Maximum Entropy (MaxEnt) framework to simulate the potential richness distribution of a comprehensive set of 321 invasive plant species across China for the current period and future periods (2050s, 2070s, 2090s) under three Shared Socioeconomic Pathways (SSP126, SSP245, SSP585). The primary objectives of this investigation are threefold: (1) to delineate current and future potential richness patterns of invasive plants at a national scale; (2) to characterize the magnitude and directionality of predicted shifts in the geographic centroid of species richness under alternative climate scenarios; and (3) to identify and quantify the principal environmental determinants governing these emergent distribution patterns. Ultimately, this study endeavors to furnish a robust scientific foundation to underpin the strategic prioritization of monitoring efforts and the implementation of proactive containment measures in regions forecast to experience heightened invasion risk (Figure 1).

## 2. Results

### 2.1. Model Performance and Key Environmental Drivers

**Figure 2:**
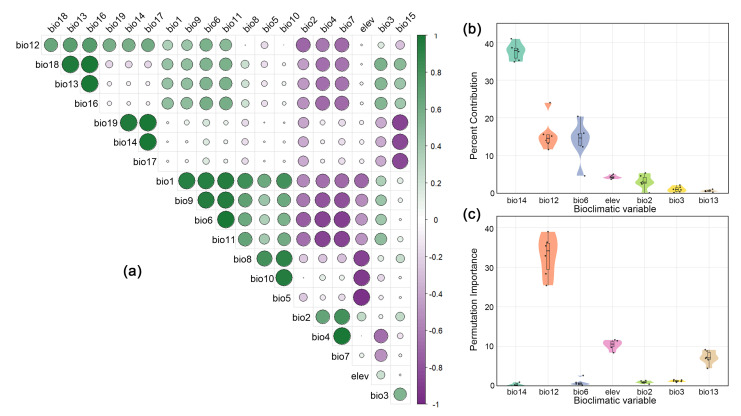
Analysis of bioclimatic variables for species distribution modeling: (**a**) correlation matrix of 20 initial bioclimatic variables; (**b**) percent contribution of the seven selected variables to the current distribution predictions; (**c**) permutation importance of the selected variables.

To mitigate the potentially confounding effects of multicollinearity on model stability and ecological inference, a Pearson correlation analysis was initially conducted across the full suite of 20 bioclimatic variables to identify pairs with high collinearity (|r| > 0.8). From each group of highly correlated variables, the single predictor demonstrating the highest percent contribution in preliminary MaxEnt model runs was retained, thereby maximizing ecological interpretability while minimizing redundancy (Figure 2a). The variables retained from collinear groups included: bio6 (Min Temperature of Coldest Month, 13.75%), bio2 (Mean Diurnal Range, 3.06%), elev (Elevation, 4.29%), bio13 (Precipitation of Wettest Month, 0.62%), and bio14 (Precipitation of Driest Month, 37.57%). In addition, bio3 (Isothermality) and bio12 (Annual Precipitation) were retained as independent predictors, given their minimal correlation (|r| ≤ 0.8) with all other selected variables and their respective contributions of 1.03% and 15.60%. Collectively, this parsimonious set of seven environmental predictors—bio2, bio3, bio6, bio12, bio13, bio14, and elev—effectively captured the principal gradients of thermal regime, moisture availability, and topography, exhibited strong statistical independence, and thereby adequately mitigated concerns of multicollinearity. Among these selected variables, bio6 (Min Temperature of Coldest Month) and bio14 (Precipitation of Driest Month) emerged as the predominant determinants of invasive plant distribution and richness patterns (Figure 2b,c).

The four most influential environmental variables were identified based on their mean percent contribution and permutation importance scores across all species models. Optimal ranges for these key variables were delineated by applying the dual criteria of a predicted occurrence probability greater than 0.5 [20] and a species richness of at least 30 species per grid cell. The resulting optimal ranges were as follows: Precipitation of Driest Month (bio14; range: 1–202 mm, mean: 24.1 mm), Minimum Temperature of Coldest Month (bio6; range: −16.83–21.27 °C, mean: 0.92 °C), Annual Precipitation (bio12; range: 254–4434 mm, mean: 1187.94 mm), and Elevation (elev; range: −3–3214 m, mean: 168.69 m). Leveraging the outcomes of systematic parameter optimization conducted using the ‘ENMeval’ package, the final ensemble of species distribution models—comprising 251 species modeled with tuned parameters and 70 species with default settings—exhibited excellent predictive performance (mean AUC = 0.972 ± 0.037; mean TSS = 0.877 ± 0.115). Sensitivity and specificity averaged 0.950 ± 0.053 and 0.926 ± 0.076, respectively. An overwhelming majority of species achieved high accuracy: 92.1% had AUC > 0.9, and 98.1% had TSS > 0.6. Examination of the empirical occurrence data revealed a markedly heterogeneous and fragmented pattern of observed invasive plant richness across the Chinese landscape. Contemporary hotspots of high species richness were predominantly concentrated south of the Taihang Mountains, notably within the hill regions of Guangdong and Guangxi, the Yangtze River Delta, the southern Fujian hills, and Hainan Island, where they exhibited a “small and scattered” patchy distribution. In contrast, the model-projected high-richness zones appeared substantially more extensive and spatially contiguous than the observed distribution. Specifically, the model anticipated that high-richness areas would form broader, more connected swaths, particularly along the coastlines of Guangdong and Taiwan, across Hainan Island, throughout the Yangtze River Delta, and over the lake plains of Hunan and Hubei provinces. Comparison of the 0.5 threshold with species-specific maxSSS thresholds further confirmed the robustness of these spatial patterns: all grid cells classified as high-richness (≥31 species) under the 0.5 threshold were also identified as high-richness under maxSSS, indicating that the core hotspot locations are insensitive to threshold choice.

### 2.2. Predicted Trends of Species Distribution Ranges Under Climate Scenarios

**Figure 3:**
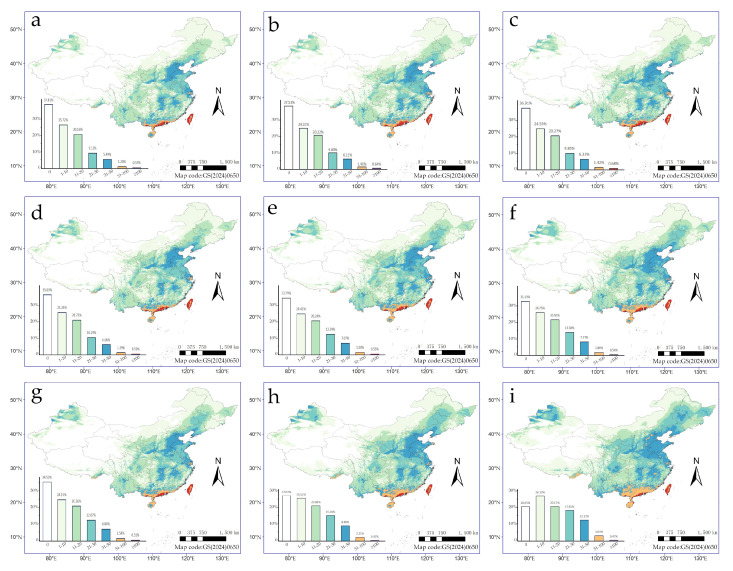
Projected spatial patterns of invasive plant species richness across China under future climate scenarios: (**a**–**c**) 2050s under SSP126, SSP245, and SSP585, respectively; (**d**–**f**) 2070s under the same three scenarios; (**g**–**i**) 2090s under the same three scenarios.

Simulations derived from the ensemble of MaxEnt models indicated that projected climate change is poised to precipitate substantial spatial reorganization of invasive plant species richness across China (Figure 3).

Relative to the current climatic baseline detailed in Table 1, regions characterized by low species richness (0–10 species) underwent consistent and significant contractions, with their total areal proportion declining from 68.52% under current conditions to 46.38% by the 2090s under the SSP585 scenario. Conversely, areas of medium and high species richness demonstrated unequivocal expansionary trends. Specifically, the extent of medium-richness areas (11–30 species) expanded steadily from 23.59% to 37.99% under SSP585-2090s. The expansion of high-richness regions (≥31 species) was particularly pronounced, with their areal coverage nearly doubling from 7.89% under the current baseline to a peak of 15.63% under the SSP585-2090s scenario.

Notably, within each SSP scenario, areas completely devoid of invasive plants (0 species) and those with low richness (1–10 species) underwent progressive, and in many cases accelerating, contraction. In contrast, areas of medium and high richness exhibited persistent expansion. Under the SSP245 scenario, for instance, both medium and high richness categories exhibited discernible expansion, with the most pronounced increases manifesting in the 2090s. In contrast, areas devoid of invasive plants (0 species) underwent continuous and substantial contraction, a trend that was especially pronounced under the SSP585 scenario. Under this high emission pathway, the proportion of such areas plummeted from 38.02% to 34.53% (2050s), 27.02% (2070s), and ultimately to 20.05% (2090s). These areas were progressively supplanted by those harboring low, medium, and high species richness, with the expansion of medium- and high-richness areas occurring at the most accelerated pace. Across all future projections, high richness areas consistently accounted for more than 7% of China’s total land area, reaching 15.63% (28,870 grid cells) under the 2090s-SSP585 scenario, representing a near doubling relative to the current baseline estimate of 7.89%. Remarkably, under each emission scenario, the proportion of high-richness areas exhibited a consistent upward trajectory across the three time horizons, from the 2050s through the 2090s. Concomitantly, medium-richness areas (11–30 species) also expanded relative to the present, albeit less dramatically, whereas low-richness areas (1–10 species) exhibited a consistent declining trend.

### 2.3. Changes in Species Richness Under Future Climatic Conditions

**Figure 4:**
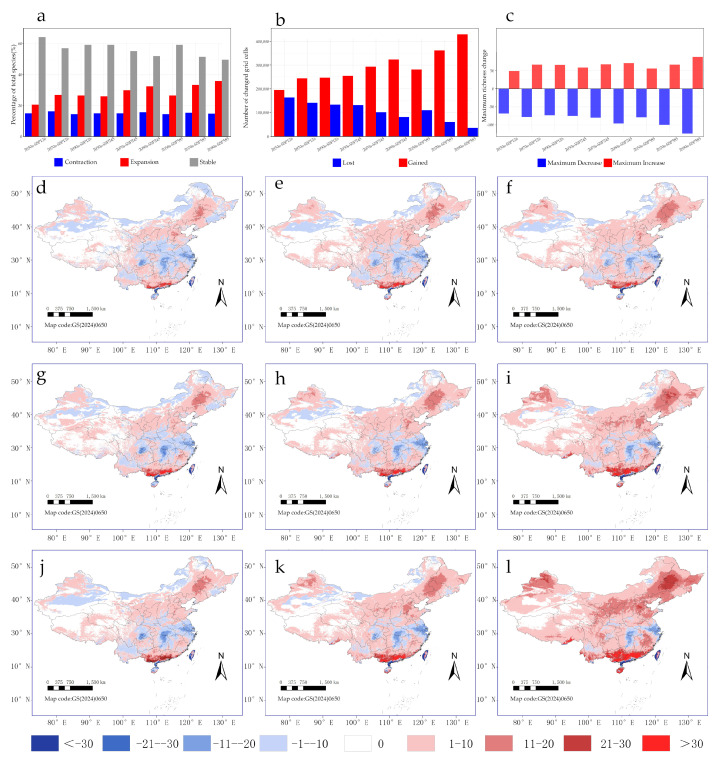
Projected changes in invasive plant richness. (**a**) Number of species exhibiting range expansion, contraction, or stability across the nine scenario–period combinations; (**b**) Number of grid cells with a net increase or net decrease in species richness; (**c**) Maximum increase and decrease in richness per grid cell; (**d**–**l**) Spatial patterns of net richness change for each scenario–period combination: (**d**) 2050s-SSP126; (**e**) 2050s-SSP245; (**f**) 2050s-SSP585; (**g**) 2070s-SSP126; (**h**) 2070s-SSP245; (**i**) 2070s-SSP585; (**j**) 2090s-SSP126; (**k**) 2090s-SSP245; (**l**) 2090s-SSP585.

By comparing current and future projected distributions on a per-grid-cell basis, we categorized the range dynamics of each invasive plant species into three discrete classes: Contraction, Expansion, and Stable (defined as a net change in occupied area of less than 0.5% (Table 2)). The results revealed that across the nine future climate–period combinations, 24 species experienced substantial habitat contraction, with a mean reduction in suitable range of −50.37% among contracting species. In contrast, 58 species exhibited pronounced range expansion under the same ensemble of future scenarios, with a mean increase in suitable habitat of 145.79%. Notably, the number of species projected to expand their ranges consistently exceeded the number of those projected to contract across all future scenario–period combinations (Figure 4a). Analysis of grid-cell-level richness change (Figure 4b) further demonstrated that the number of grid cells experiencing net gains in species richness consistently exceeded the number undergoing net losses across all scenarios examined. Examination of the extreme values of richness changes (Figure 4c) revealed a consistent asymmetry: across all periods, the maximum potential decrease in richness far exceeded the maximum potential increase. Notably, the most extreme fluctuations in local richness were projected by the 2090s under SSP5-8.5, with a single grid cell gaining up to 88 species or losing up to 124. The maximum loss occurred in a currently species-rich area (229 species) in Taiwan, where the scenario entails a substantial reduction in dry-season precipitation (bio14: −17.1 mm) and a marked increase in winter minimum temperatures (bio06: +3.66 °C), despite a slight increase in annual precipitation. Such values represent the upper bound of climatic suitability loss and should not be interpreted as a prediction of near-complete local extinction. Spatial visualization of these net richness changes revealed pronounced geographic patterning (Figure 4d–l). Regions projected to accrue additional invasive species were predominantly located in the hill regions of Guangdong and Guangxi, the Northeast China Plain, and the Junggar Basin. Conversely, areas forecast to lose invasive species richness were primarily concentrated on the Leizhou Peninsula, Taiwan, in the Yangtze River Delta, and in the lake plains of Hunan and Hubei provinces.

**Figure 5:**
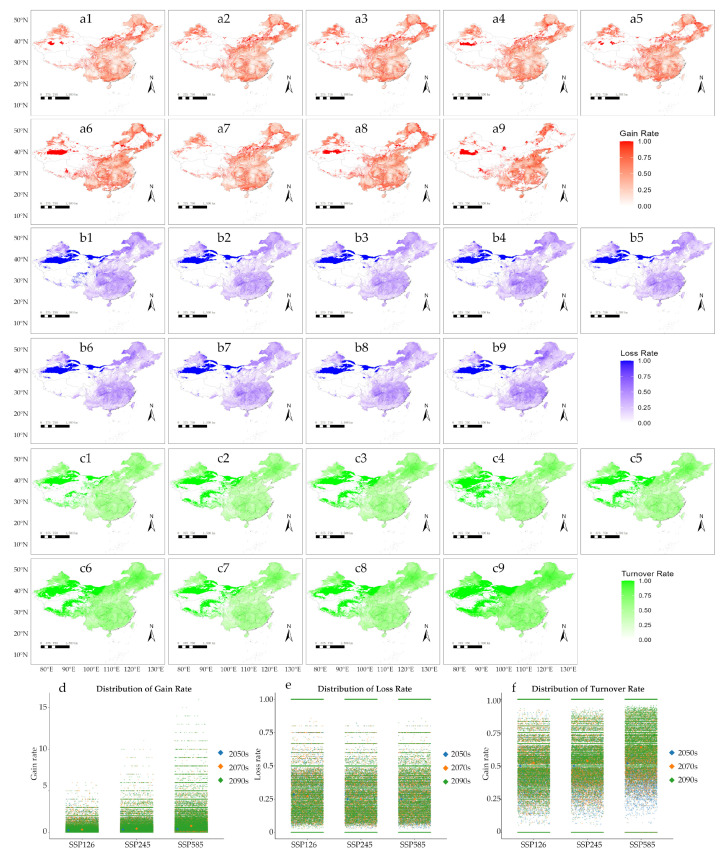
Grid-cell metrics of species compositional change under future climatic conditions. (**a1**–**a9**) Percentage of species gained; (**b1**–**b9**) Percentage of species lost; (**c1**–**c9**) species turnover value. (**d**–**f**) Distribution of richness change rates across scenarios: (1) 2050s-SSP126; (2) 2050s-SSP245; (3) 2050s-SSP585; (4) 2070s-SSP126; (5) 2070s-SSP245; (6) 2070s-SSP585; (7) 2090s-SSP126; (8) 2090s-SSP245; (9) 2090s-SSP585.

Statistical analysis of species gain rate, loss rate, and turnover rate at the grid-cell level indicated that, under future climate change, the species gain rate fell predominantly within the range of 0.01 to 1.00. Under the SSP126 scenario for the 2050s, a particularly high concentration of grid cells exhibited gain rates between 0.10 and 0.50. Likewise, grid cells with gain rates ranging from 0.10 to 0.50 were most prevalent by the 2090s under the SSP585 scenario (Figure 5d). The distribution of species turnover rates displayed a consistent upward trajectory across successive time periods, with the majority of values falling between 0.21 and 0.60. For instance, by the 2050s under the SSP126 scenario, the number of grid cells exhibiting turnover rates between 0.40 and 0.50 peaked at 111,231. Over time and with intensifying emission scenarios, the modal turnover rate interval shifted upward, from 0.40–0.50 to 0.50–0.60 (Figure 5f). Most strikingly, under the SSP585 scenario by the 2090s, there was a pronounced surge in the number of grid cells exhibiting turnover rates in the 0.60–0.90 range, and a remarkable 122,487 grid cells surpassed a turnover rate of 0.90. This indicates a near-complete compositional upheaval of invasive plant assemblages under this high-emission, end-of-century scenario (Figure 5a1–c9).

Overall, the distribution patterns of gain, loss, and turnover rates exhibited broadly consistent trends across the range of climate scenarios examined. With progressive climate forcing of higher greenhouse gas concentrations and advancing time horizons, the number of grid cells falling into high-value intervals for both gain rate and turnover rate increased markedly, underscoring the profound impact of projected climate change on species compositional dynamics. Compositional turnover culminated under the SSP585 scenario by the 2090s. This pattern aligns closely with the elevated richness gains projected for this high-emission pathway, thereby highlighting the substantial ecological disruption risks confronting ecosystems under unmitigated climate forcing. Specifically, the mean turnover rate across all grid cells under SSP585 by the 2090s reached 57.4%, indicating that, on average, nearly three-fifths of the local invasive species assemblage is projected to be replaced. This pronounced turnover was driven primarily by the exceptionally high mean species gain rate per grid cell under this scenario, which reached 66.1%, a finding fully consistent with the observed patterns of richness augmentation.

**Figure 6:**
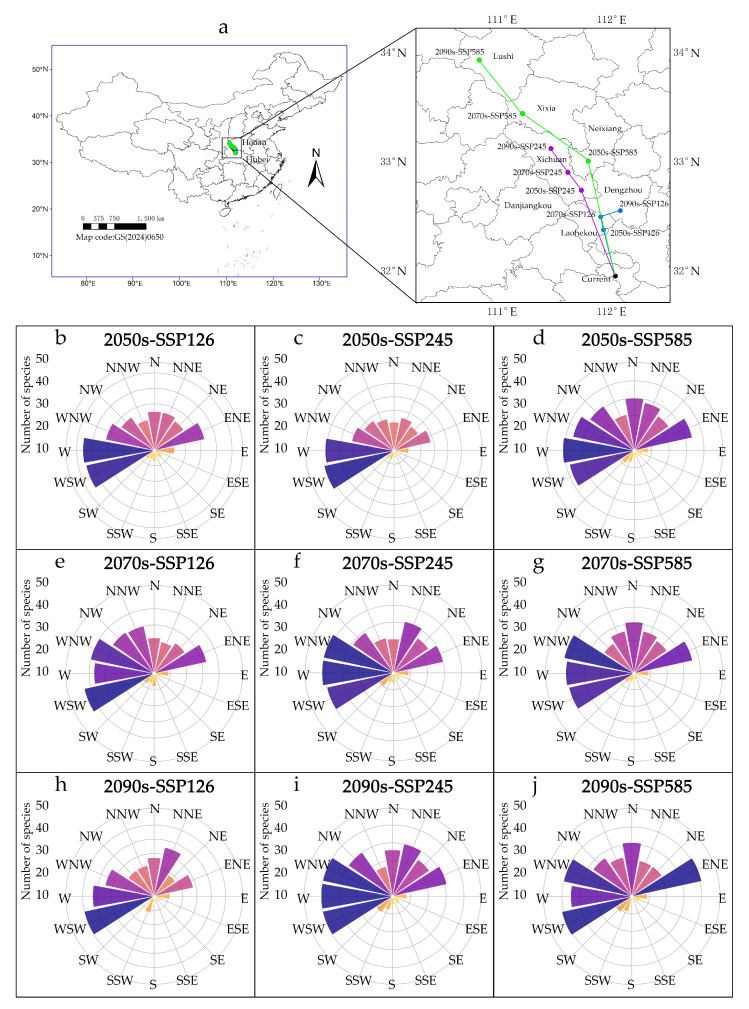
Geographic shifts in the centroid of invasive plant species richness under future climate scenarios: (**a**) movement trajectory of the richness centroid from current to 2090s under different SSPs; (**b**–**j**) direction and distance of centroid shifts for each scenario–period combination relative to the current baseline (color intensity indicates migration distance).

As climate change unfolds across the 21st century, this centroid is projected to migrate progressively north-northwestward, eventually entering Henan Province (Figure 6a). Collectively, our projections indicate that future climate change will drive a predominant north-northwestward displacement of the geographic centroid of invasive plant species richness (Figure 6b–j). Under the current climatic conditions, the centroid of invasive plant species richness is located in Xiangcheng District, Hubei Province.

Under the SSP126 and SSP245 scenarios, the centroid is projected to shift into Henan Province, relocating to Dengzhou City and Nanyang City, respectively, within Jingzhou County. In contrast, under the SSP585 scenario, the magnitude of this north-northwestward displacement is substantially greater. By the 2090s under SSP585, the centroid is anticipated to migrate northwestward to Lushi County, within the prefecture-level city of Sanmenxia, Henan Province. Analysis of the environmental attributes associated with the shifting richness centroid revealed a consistent trajectory toward conditions of higher elevation and increased precipitation (Table 3).

## 3. Discussion

### 3.1. Drivers of Model Performance and Richness Patterns

The ensemble of MaxEnt models developed in this study demonstrated exceptional predictive performance (mean AUC = 0.972 ± 0.037; mean TSS = 0.877 ± 0.115), with 92.1% of species achieving AUC > 0.9 and 98.1% achieving TSS > 0.6, thereby confirming the robustness and reliability of the projected richness patterns. Consistent with findings from previous large-scale assessments, our results indicate that precipitation-related variables-most notably precipitation of the driest month (Bio14, 37.57%) and annual precipitation (Bio12)-exerted a substantially stronger constraint on invasive plant distributions than did temperature-related factors.

This finding underscores the critical role of water availability as a primary limiting factor governing the establishment and proliferation of alien plant species, a pattern consistent with China’s monsoon climate, where the pronounced spatial and seasonal heterogeneity of precipitation imposes a stronger biogeographic filter than the more gradually varying thermal regime. This constraint is likely to be exacerbated by projected increases in drought frequency and intensity under future climate scenarios [21].

The pronounced importance of the minimum temperature of the coldest month (Bio6) further highlights the potential for poleward and upward range expansions as winter thermal constraints relax under a warming climate. Current climate projections indicate a global expansion of hotter and drier climates, which may facilitate the establishment of invasive annual plants by enabling them to employ both drought escape and drought resistance strategies to colonize novel semi-arid environments [22]. Moreover, elevated temperatures have been shown to directly facilitate the naturalization success of alien herbaceous species [23]. As minimum temperatures rise and the frequency of frost events diminishes, the poleward spread of invasive species becomes increasingly physiologically feasible [24]. Additionally, elevation exerts an indirect influence on species distributions by modulating both thermal and hydrological regimes. Consequently, under future high-emission scenarios, invasive plant assemblages may undergo upward elevational shifts in search of analogous climatic niches.

### 3.2. Richness Distribution Patterns and Future Shifts

By simulating richness suitability surfaces under both current and projected future climates, our study generates actionable, species-specific lists that identify those invasive taxa with the greatest potential for future range expansion. Such information enables natural resource managers to proactively prioritize geographic areas where invasive species are forecast to attain high abundance and exert disproportionate ecological or economic impacts [25]. In summary, projected climate change is anticipated to precipitate a profound spatial reorganization of invasive plant species richness across China. Relative to current conditions, areas characterized by low species richness are projected to undergo continued contraction, whereas those of medium and high richness are forecast to expand considerably. This divergence is most pronounced under the high-emission SSP5-8.5 pathway, under which the areal proportion of high-richness zones (≥31 species) nearly doubles, increasing from 7.9% to 15.6%. For those species projected to experience expanding habitat suitability in specific regions, targeted management interventions should focus on either preemptive monitoring of range-shifting invasions or the strategic eradication of nascent satellite populations [26]. The projected north-northwestward migration of the species richness centroid represents a coherent, large-scale biogeographic response of invasive plant assemblages to anticipated climatic shifts. This directional migration pattern is likely driven by a confluence of factors, most notably projected increases in precipitation and the amelioration of winter thermal constraints across northwestern China. Notably, the most dramatic compositional turnover was projected for the 2090s under the SSP585 scenario, with the mean turnover rate across all changing grid cells reaching 72.9%. This finding highlights the severe ecological disruption risks confronting ecosystems under unmitigated, high-concentration emission pathways. Phenological shifts induced by climate warming—for instance, the advancement of flowering by an average of 1.73 days per degree Celsius of warming—may extend the pre-reproductive period and thereby contribute mechanistically to the elevated species turnover rates observed in our projections [27].

### 3.3. Management Implications

The findings of this study have direct and actionable implications for invasive species policy and management. We propose a three-pronged strategic framework: (1) Augment monitoring and early-warning surveillance in geographic locales forecast to undergo substantial increases in invasion pressure, most notably the Northeast China Plain, the Junggar Basin, and the hill regions of Guangdong and Guangxi; (2) Establish strategic containment and control buffer zones along predicted migration corridors—with particular emphasis on the Dabie Mountains ecotone straddling the Hubei–Henan provincial border—to intercept range-expanding populations; and (3) Integrate ecological restoration principles with invasive species management, for instance by promoting competitive native species assemblages that can effectively suppress invasive populations, a strategy that may yield synergistic conservation benefits [28].

### 3.4. Limitations and Future Research Directions

While the study provides a comprehensive, spatially explicit assessment of climate-driven shifts in invasive plant richness across China, several limitations warrant consideration. First, the stacked MaxEnt framework assumes that climatic suitability equates to species occurrence, implicitly treating the ‘M’ and ‘B’ components of the BAM diagram as fully overlapping. In reality, non-climatic factors—such as propagule pressure, human-mediated dispersal, transportation networks, land-use change, soil properties, and disturbance regimes—strongly mediate whether a climatically suitable area is actually occupied. Our projections should therefore be interpreted as maps of potential invasion risk rather than deterministic forecasts of future distributions. Second, although an ensemble of seven GCMs was used to reduce inter-model uncertainty, structural differences among climate models, particularly in precipitation projections, remain a source of irreducible uncertainty. Third, species occurrence data were refined to the county level and mapped to 2.5 arc-minute grid cells, potentially overestimating spatial continuity in complex terrain. Fourth, the binary threshold of 0.5 used to convert continuous suitability into presence–absence maps, though common, can influence richness and turnover estimates; future work could adopt species-specific thresholds (e.g., maxSSS) to improve accuracy. Fifth, extreme grid-level richness changes—such as the maximum loss of 124 species observed in Taiwan under SSP5-8.5—partly reflect the extrapolation of climatic envelopes under intensified drought seasonality and should be regarded as upper-bound estimates of climate-driven risk, as they do not account for species’ capacities for in situ adaptation or microhabitat buffering. Finally, the 321 modeled species are predominantly widespread or already naturalized invaders; rare or incipient invaders with high spread potential may be underrepresented. Future research should integrate dispersal constraints, land-use and soil variables, and multi-model ensemble platforms (e.g., biomod2) to better partition uncertainty and explore responses by functional groups to capture guild-specific dynamics. Such advances will be essential for translating climatic suitability projections into operational biosecurity guidance.

## 4. Conclusions

This study provides a comprehensive, high-resolution assessment of the potential shifts in invasive plant species richness across China under three alternative Shared Socioeconomic Pathways. Our projections reveal a coherent and concerning pattern: a pronounced north-northwestward displacement of the species richness centroid, a near doubling of high-richness areas (from 7.9% to 15.6% under SSP585 by the 2090s), and a dramatic acceleration of species compositional turnover under high-emission scenarios. These findings underscore the heightened invasion risks confronting northwestern and northeastern China and highlight the urgent need for spatially targeted monitoring and adaptive management strategies.

To transcend the inherent limitations of the correlative modeling approach employed herein, future research should pivot toward the implementation of integrated ensemble modeling platforms, such as biomod2, to better quantify and partition model-based uncertainty. Furthermore, simulations stratified by plant functional groups or life-history strategies (e.g., annuals, perennials, shrubs) are warranted to elucidate the distinct response mechanisms of different ecological guilds to climatic forcing. The incorporation of advanced artificial intelligence techniques—including deep-learning architectures and graph neural networks—holds considerable promise for disentangling the complex, nonlinear relationships that characterize ecological systems and for improving the predictive capacity of species distribution models. Concurrently, the synergistic integration of high-resolution remotely sensed data (e.g., from unmanned aerial vehicles) with systematic field monitoring programs will be instrumental in constructing multi-source, high-fidelity species distribution databases. Such databases are essential for providing the robust, spatially explicit scientific support required for the precise prevention, strategic control, and adaptive governance of China’s invasive flora in an era of accelerating global change. This study thus provides a timely and spatially explicit framework to guide national biosecurity efforts.

## 5. Materials and Methods

### 5.1. Species Distribution Data

**Figure 7:**
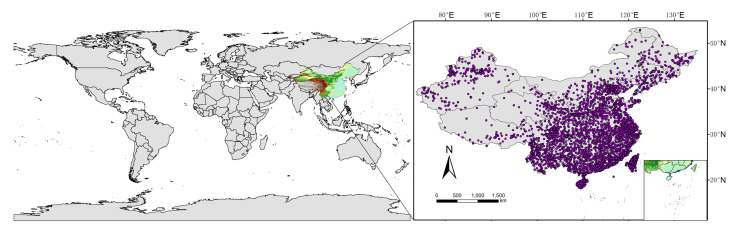
Geographic extent of the study area and the documented distribution of invasive alien plant species across China. The map depicts the actual (observed) distribution based on the curated occurrence dataset. Cartography was based on the 2024 edition of the Standard Map of China [Approval Number: GS (2024)0650], released by the Ministry of Natural Resources of China and downloaded from the National Platform for Common Geo-spatial Information Services.

Species occurrence data were primarily sourced from the most comprehensive and highest-resolution county-level database of alien invasive plants currently available for China [29]. In this database, each occurrence record is georeferenced with geographic coordinates, while the county designation provides an administrative attribute rather than the spatial grain of the record. Geographic occurrence records for all species were compiled from multiple repositories, including the Chinese Virtual Herbarium (CVH), national and regional biodiversity databases, published floras, and the Global Biodiversity Information Facility (GBIF). All records were subjected to rigorous quality control procedures and subsequently refined to the county level. Following this initial compilation and quality assurance, the raw dataset comprised occurrence records for 373 species, spanning 238 genera and 92 families, totaling 172,351 individual records. To ensure data quality and model robustness, we subsequently filtered the raw dataset by removing duplicate records, occurrences falling outside the geographic boundaries of China, and any species represented by fewer than five unique occurrence points [30]. After applying these filtering criteria, the final curated dataset retained for modeling consisted of 167,359 occurrence records, representing 321 plant species distributed across 196 genera and 63 families (Figure 7). The most species-rich families were *Asteraceae* (58 species), *Fabaceae* (37 species), and *Poaceae* (19 species). This filtering aimed to improve model stability by excluding species with insufficient training data. Consequently, the final dataset mainly comprises relatively widespread or naturalized invaders, and the resulting projections best reflect the climate responses of established alien species. Given that the fidelity of model projections is contingent upon both the quality of species occurrence data and the spatial resolution of environmental predictors, the study domain (China) was discretized into a grid comprising 553,930 cells at a spatial resolution of 2.5 arc-minutes (approximately 0.041666667° at the equator). Species richness was subsequently calculated for each grid cell by summing the number of species projected to occur therein.

### 5.2. Environmental Variables

Climate data for both the baseline and future periods were obtained from the WorldClim v2.1 database [31]. The current climate dataset comprised 19 bioclimatic variables representing average conditions for the period 1970–2000, while future climate data were derived from the Coupled Model Intercomparison Project Phase 6 (CMIP6) under three Shared Socioeconomic Pathway (SSP) scenarios: SSP126 (sustainability pathway), SSP245 (intermediate pathway), and SSP585 (fossil-fueled development pathway) [32]. To assess long-term climate trends and minimize the influence of interannual variability, 20-year climatological averages were computed for three consecutive future periods: 2041–2060 (2050s), 2061–2080 (2070s), 2081–2100 (2090s). To enhance the robustness of future climate projections and to account for inter-model uncertainty, outputs from an ensemble of seven global climate models participating in CMIP6 were integrated [20]. These models included BCC-CSM2-MR, MIROC6, IPSL-CM6A-LR, MRI-ESM2-0, EC-Earth3-Veg, INM-CM5-0 and CMCC-ESM2. They were selected to span a range of equilibrium climate sensitivities and to ensure complete data availability across all three SSPs and time periods in the WorldClim archive. All environmental layers were resampled to a uniform spatial resolution of 2.5 arc-minutes and subsequently clipped to the terrestrial boundaries of China using a national vector map [33]. An ensemble mean future climate dataset was subsequently generated by calculating the arithmetic mean of the seven GCM outputs for each of the 19 bioclimatic variables, per SSP scenario and time period, yielding a single consensus raster layer for each variable across the study area. Extraction of the 19 bioclimatic variables at the target 2.5 arc-minute resolution for current and future periods was facilitated using a 1:14 million-scale vector map of China. In addition to the bioclimatic variables, elevation data (elev) derived from the WorldClim 2.1 database were incorporated as a topographical predictor.

To mitigate the potentially adverse effects of multicollinearity on model stability and parameter interpretability [34], a Pearson correlation analysis was performed on the full suite of 19 climate variables using values extracted at all invasive plant occurrence points. A pairwise linear correlation matrix was constructed to identify highly correlated variable pairs indicative of potential collinearity. Subsequently, the Variance Inflation Factor (VIF) was calculated for each variable to provide a quantitative assessment of the severity of multicollinearity [35]. Finally, from each pair of variables exhibiting a Pearson correlation coefficient |r| > 0.8, a single predictor was selected for retention based on its ecological relevance and contribution to the MaxEnt models [19,36].

### 5.3. Species Distribution Model

Species distribution modeling was conducted using the maximum entropy algorithm implemented in MaxEnt v3.4.0. For model training and evaluation, the occurrence dataset for each species was randomly partitioned into training (75%) and validation (25%) subsets [37]. Model uncertainty was assessed using a bootstrap resampling procedure with 10 replicate runs. For each replicate, 10,000 background points were randomly sampled from the study area, and the optimization algorithm was permitted a maximum of 500 iterations to ensure convergence [38]. To optimize model complexity and avoid overfitting, systematic parameter tuning was performed using the ENMeval v2.0.4 package in R 4.3.2. The tuning procedure explored a parameter space encompassing six feature class combinations (L, LQ, H, LQH, LQHP, LQHPT) and a sequence of regularization multipliers ranging from 0.1 to 4.0, incremented by 0.2 [39]. Model performance during tuning was evaluated using the Area Under the Receiver Operating Characteristic Curve (AUC) and the corrected Akaike Information Criterion (AICc), which balances model fit against complexity [40]. For each species, the parameter combination that minimized the AICc value was selected as the optimal model configuration. Final models were run with 10 bootstrap replicates, with the output format set to logistic (yielding a continuous probability surface ranging from 0 to 1). Default settings were retained for all other parameters [41]. After model construction, we additionally assessed predictive performance by calculating the True Skill Statistic (TSS), sensitivity, and specificity for each species based on the maximum training sensitivity plus specificity threshold. Following widely adopted conventions, AUC values exceeding 0.9 were considered indicative of high accuracy, and TSS values exceeding 0.6 were regarded as indicative of good discriminatory ability [42,43].

To evaluate the relative contributions of the selected environmental predictors to the species distribution models, a jackknife test was conducted to assess variable importance [44]. The average species occurrence probability, ranging from 0 to 1, for each grid cell was computed by averaging the logistic output across the 10 bootstrap replicate simulations [45].

### 5.4. Data Analysis

To systematically assess species-specific range responses to projected climate change, the continuous habitat suitability predictions were transformed into binary presence–absence maps by applying a threshold of 0.5. This moderate cutoff was adopted to balance omission and commission errors across species with widely varying prevalence and is consistent with prior multi-species syntheses [41,46]. Sensitivity tests using species-specific maxSSS thresholds confirmed that the spatial patterns of richness hotspots are robust to threshold choice. This binarization procedure enabled the categorization of grid-cell-level habitat changes into three discrete classes: gain (0 to 1), loss (1 to 0), and retention (1 to 1). Based on this classification, two key metrics were quantified for each species: (i) the net change in suitable habitat area, and (ii) the categorical range dynamics. The net change in suitable habitat area reflects the magnitude of range expansion (positive values) or contraction (negative values) experienced by a species [47]. The categorical range dynamics classification assigned each species to one of three response modes-Expansion, Contraction, or Stable-based on whether the proportional change in suitable area exceeded a tolerance threshold of ±0.5% [48,49]. Spatial patterns of species richness for both the current period and each future scenario–period combination were derived by summing the binary presence–absence maps across all 321 species. Net changes in species richness were subsequently determined by subtracting the baseline richness map from each future richness projection. Positive differences in values denote local increases in species richness, whereas negative values indicate local decreases. Finally, to further elucidate the dynamic characteristics of projected species compositional shifts, we calculated three grid-cell-level metrics: species gain rate, species loss rate, and species turnover rate.

To assess the potential directionality and magnitude of range shifts under future climate scenarios, we quantified the displacement of the geographic centroid of each species’ projected distribution relative to the baseline climate. Specifically, the geographic centroid of invasive plant species richness was calculated for each time period by weighting the spatial coordinates of each grid cell by its respective species richness value. Subsequently, the great-circle distance formula was employed to compute the straight-line distance between centroids for consecutive time steps, and the direction of migration was derived from the azimuth angle. This allowed for a spatially explicit characterization of the continuous migration trajectory from the baseline period to each future time horizon [34].

## Figures and Tables

**Table 1:** Number and percentage of grid cells classified into species richness categories under current and future climate scenarios (2050s, 2070s, 2090s) across three SSPs (SSP126, SSP245, SSP585).

Species Richness			0	1–10	11–20	21–30	31–50	51–100	≥100	Max
Current		Number	210,607	168,930	82,028	48,641	32,443	8043	3238	243
		Percentage	38.02%	30.50%	14.81%	8.78%	5.86%	1.45%	0.58%	
2050s	SSP126	Number	209,432	142,165	111,672	50,447	30,436	6639	3139	220
		Percentage	37.81%	25.72%	20.16%	9.11%	5.49%	1.2%	0.57%	
	SSP245	Number	197,691	139,886	114,810	56,982	33,555	7711	3295	215
		Percentage	35.69%	25.25%	20.73%	10.29%	6.06%	1.39%	0.59%	
	SSP585	Number	191,257	133,757	112,773	66,879	37,895	8523	2846	207
		Percentage	34.53%	24.15%	20.36%	12.07%	6.84%	1.54%	0.51%	
2070s	SSP126	Number	207,885	134,654	112,013	54,267	33,847	7735	3529	216
		Percentage	37.53%	24.31%	20.22%	9.8%	6.11%	1.4%	0.64%	
	SSP245	Number	187,154	135,319	112,123	68,335	39,181	8755	3063	209
		Percentage	33.79%	24.43%	20.24%	12.34%	7.07%	1.58%	0.55%	
	SSP585	Number	149,663	141,378	115,677	83,142	49,232	12,227	2611	196
		Percentage	27.02%	25.52%	20.88%	15.01%	8.89%	2.21%	0.47%	
2090s	SSP126	Number	204,469	135,996	112,267	54,536	35,039	7872	3751	220
		Percentage	36.91%	24.55%	20.27%	9.85%	6.33%	1.42%	0.68%	
	SSP245	Number	172,747	137,119	113,581	74,112	43,042	10,311	3018	195
		Percentage	31.19%	24.75%	20.5%	13.38%	7.77%	1.86%	0.54%	
	SSP585	Number	111,063	145,835	111,734	98,688	67,155	16,837	2618	195
		Percentage	20.05%	26.33%	20.17%	17.82%	12.12%	3.04%	0.47%	

**Table 2:** Mean species gain rate (%), loss rate (%), and turnover rate for all grid cells exhibiting changes in species richness under alternative future climate scenarios.

Period	2050s			2070s			2090s		
Climate scenarios	SSP126	SSP245	SSP585	SSP126	SSP245	SSP585	SSP126	SSP245	SSP585
Mean Gain Rate (%)	41.34	51.18	63.70	49.30	68.72	117.77	48.16	88.83	191.96
Mean Loss Rate (%)	33.48	29.49	29.61	32.50	29.90	30.61	30.51	30.48	31.87
Mean Turnover Rate (%)	51.94	53.39	55.85	55.41	57.32	65.26	53.35	61.15	72.89

**Table 3:** Values of the seven key environmental variables at the centroid of maximum species richness under current and future climate scenarios.

Variable	Current	2050s			2070s			2090s		
		SSP126	SSP245	SSP585	SSP126	SSP245	SSP585	SSP126	SSP245	SSP585
bio02 (°C)	9.15	9.81	10.67	10.51	10.11	10.26	10.97	10.49	10.23	10.57
bio03 (°C)	27.86	29.19	31.43	30.73	30	30.5	31.71	30.36	30.79	31.11
bio06 (°C)	−1.37	−0.1	−0.07	0.13	0.11	0.93	0.86	−0.34	1.69	0.37
bio12 (mm)	977	1110.71	1060.71	1037.57	1093.71	1039.85	912	1080	1013.71	868.14
bio13 (mm)	175	199	187.14	185.29	193.57	184.29	174.14	192.71	187.29	185.86
bio14 (mm)	16	15.57	14.71	14	15.71	14	8.57	16.43	11.71	8
Elev (m)	107	133	180	277	129	250	469	119	250	947

## Data Availability

The data used in this study were derived from a comprehensive database, and were not directly accessed or downloaded from the GBIF network. The data and code that support the findings of this study are openly available in Zenodo at https://doi.org/10.5281/zenodo.18320432. Species occurrence data were obtained from the comprehensive county-level distribution database of alien and invasive plants in China at https://doi.org/10.1002/ecy.70084. Climate and elevation data were obtained from WorldClim 2.1 at https://www.worldclim.org. No specific permits were required for plant occurrence data collection. The original contributions presented in the study are included in the article; further inquiries can be directed to the corresponding authors.

## References

[1] Macdonald J.S., Lutscher F., Bourgault Y. (2024). Climate change fluctuations can increase population abundance and range size. Ecol. Lett..

[2] Bellard C., Thuiller W., Leroy B., Genovesi P., Bakkenes M., Courchamp F. (2013). Will climate change promote future invasions?. Glob. Change Biol..

[3] Pecl G.T., Araújo M.B., Bell J.D., Blanchard J., Bonebrake T.C., Chen I.C., Clark T.D., Colwell R.K., Danielsen F., Evengård B. (2017). Biodiversity redistribution under climate change: Impacts on ecosystems and human well-being. Science.

[4] Diagne C., Leroy B., Vaissière A.C., Gozlan R.E., Roiz D., Jaric I., Salles J.M., Bradshaw C.J.A., Courchamp F. (2021). High and rising economic costs of biological invasions worldwide. Nature.

[5] Wynne J.J., Howarth F.G., Mammola S., Ferreira R.L., Cardoso P., Di Lorenzo T., Galassi D.M.P., Medellin R.A., Miller B.W., Sanchez-Fernández D. (2021). A conservation roadmap for the subterranean biome. Conserv. Lett..

[6] Rincón V., Velázquez J., Gutiérrez J., Hernando A., Khoroshev A., Gómez I., Herráez F., Sánchez B., Luque J.P., García-abril A. (2021). Proposal of new Natura 2000 network boundaries in Spain based on the value of importance for biodiversity and connectivity analysis for its improvement. Ecol. Indic..

[7] Dinerstein E., Joshi A.R., Vynne C., Lee A.T.L., Pharand-Deschênes F., França M., Fernando S., Birch T., Burkart K., Asner G.P. (2020). A “Global Safety Net” to reverse biodiversity loss and stabilize Earth’s climate. Sci. Adv..

[8] Aguirre-Gutiérrez J., Díaz S., Rifai S.W., Corral-Rivas J.J., Nava-Miranda M.G., González-M R., Hurtado-M A.B., Revilla N.S., Vilanova E., Almeida E. (2025). Tropical forests in the Americas are changing too slowly to track climate change. Science.

[9] van Kleunen M., Dawson W., Essl F., Pergl J., Winter M., Weber E., Kreft H., Weigelt P., Kartesz J., Nishino M. (2015). Global exchange and accumulation of non-native plants. Nature.

[10] Hao Q., Ma J.S. (2023). Invasive alien plants in China: An update. Plant Divers..

[11] Wang P.L., Xue Y.T., Jiang H.B., Liu C.L., Zhang C., Huang H.K., Zhang G.F., Wan F.H., Zhang Y.B., Courchamp F. (2025). Escalating economic costs of invasive species in China driven by hidden impacts and policy gaps. Entomol. Gen..

[12] Gianoli E., Molina-Montenegro M.A. (2021). Evolution of physiological performance in invasive plants under climate change. Evolution.

[13] Phillips S.J., Anderson R.P., Dudík M., Schapire R.E., Blair M.E. (2017). Opening the black box: An open-source release of Maxent. Ecography.

[14] Faure J.P.B., Drouilly M., Botha A.E., Ross M.D., Spalton J.A., Alhlafi M., Dunford C.E., Mills D.R., De Bruin R., Gallacher E. (2024). Blanford’s fox (*Vulpes cana*) habitat suitability in Saudi Arabia: Insights from camera trapping and ensemble species distribution modelling. J. Arid Environ..

[15] Lin M., Ye X.Z., Zhao Z.X., Chen S.P., Liu B. (2025). Comparative Analysis of Habitat Expansion Mechanisms for Four Invasive *Amaranthaceae* Plants Under Current and Future Climates Using MaxEnt. Plants.

[16] Khwarahm N.R. (2025). MaxEnt-Based Distribution Modeling of the Invasive Species *Phragmites australis* Under Climate Change Conditions in Iraq. Plants.

[17] Ren W.J., Peng J., Shrestha N., Bian Z.H., Yang Y.B., Liu J.Q., Liu X., Huang P., Wu J.H. (2025). Potential distribution and future shifts of invasive alien plants in China under climate change. Glob. Ecol. Conserv..

[18] Shen S.C., Zheng F.P., Zhang W., Xu G.F., Li D.Y., Yang S.S., Jin G.M., Clements D.R., Nikkel E., Chen A.D. (2024). Potential distribution and ecological impacts of *Acmella radicans* (Jacquin) RK Jansen (a new Yunnan invasive species record) in China. BMC Plant Biol..

[19] Zhang Z., Gao W.Q., Lei X.D., Sun J.J. (2025). Range shifts of four *Larix* species across a three-dimensional geographic gradient in response to climate change. J. For. Res..

[20] Yang Q.Y., Ye X.Z., Guo G.H., Li L. (2025). Effects of climate change on the richness distribution of *Phyllostachys* species in China. J. For. Res..

[21] Garcia R.A., Cabeza M., Rahbek C., Araújo M.B. (2014). Multiple Dimensions of Climate Change and Their Implications for Biodiversity. Science.

[22] Welles S.R., Funk J.L. (2021). Patterns of intraspecific trait variation along an aridity gradient suggest both drought escape and drought tolerance strategies in an invasive herb. Ann. Bot..

[23] Haeuser E., Dawson W., van Kleunen M. (2019). Introduced garden plants are strong competitors of native and alien residents under simulated climate change. J. Ecol..

[24] Sheppard C.S., Burns B.R., Stanley M.C. (2014). Predicting plant invasions under climate change: Are species distribution models validated by field trials?. Glob. Change Biol..

[25] O’Neill M.W., Bradley B.A., Allen J.M. (2021). Hotspots of invasive plant abundance are geographically distinct from hotspots of establishment. Biol. Invasions.

[26] Spear M.J., Walsh J.R., Ricciardi A., Vander Zanden M.J. (2021). The Invasion Ecology of Sleeper Populations: Prevalence, Persistence, and Abrupt Shifts. Bioscience.

[27] Bai L., Tian L., Ren Z.G., Song X.H., Yu K.L., Meng L., Hou Z.F., Ren H.Y. (2024). Climate warming advances plant reproductive phenology in China’s northern grasslands. J. Plant Ecol..

[28] Farrell H.L., Funk J., Law D., Gornish E.S. (2022). Impacts of drought and native grass competition on buffelgrass (*Pennisetum ciliare*). Biol. Invasions.

[29] Yang Y.B., Liu X., Wu J.H., Svenning J.C., Liu J.Q., Shrestha N. (2025). A comprehensive county-level distribution database of alien and invasive plants in China. Ecology.

[30] Yang H.X., Zhang H.Y., Wang Y.R., Jia X., Hao L., Jin K., Song J. (2025). Urban bird diversity conservation plan based on the MaxEnt model and InVEST model: A case study of Jinan, China. Ecol. Indic..

[31] Farooq S. (2025). Climate-driven spread of giant hogweed *Heracleum mantegazzianum* (Sommier & Levier) in Turkey: Assessing future invasion risks under CMIP6 climate projections. BMC Plant Biol..

[32] Riahi K., van Vuuren D.P., Kriegler E., Edmonds J., O’Neill B.C., Fujimori S., Bauer N., Calvin K., Dellink R., Fricko O. (2017). The Shared Socioeconomic Pathways and their energy, land use, and greenhouse gas emissions implications: An overview. Glob. Environ. Change-Hum. Policy Dimens..

[33] Liu B., Lin M., Liu S.Y., Ye X.Z., Chen S.P. (2026). National-Scale Conservation Gaps and Priority Areas for Invasive Plant Control in China: An Integrated MaxEnt-InVEST Framework. Plants.

[34] Sun S.X., Zhang Y., Huang D.Z., Wang H., Cao Q., Fan P.X., Yang N., Zheng P.M., Wang R.Q. (2020). The effect of climate change on the richness distribution pattern of oaks (*Quercus* L.) in China. Sci. Total Environ..

[35] Dormann C.F., Elith J., Bacher S., Buchmann C., Carl G., Carré G., Marquéz J.R.G., Gruber B., Lafourcade B., Leitao P.J. (2013). Collinearity: A review of methods to deal with it and a simulation study evaluating their performance. Ecography.

[36] Xie C.P., Chen Z.Q., Yu M.T., Jim C.Y. (2025). Impact of Climate Change on the Invasion of *Mikania micrantha* Kunth in China: Predicting Future Distribution Using MaxEnt Modeling. Plants.

[37] Ma D.L., Lu Z.K., Xue Z.Q., Yu Z.H., Duan X.H., Gu X., Yao Y.K., Cai L., Zheng K.Y. (2025). Assessment of suitable habitat of *Semen Armeniacae Amarum*. in China under different climatic conditions by Internal Transcribed Spacer 2 and Maxent model. BMC Plant Biol..

[38] Yan H.Y., Feng L., Zhao Y.F., Feng L., Zhu C.P., Qu Y.F., Wang H.Q. (2020). Predicting the potential distribution of an invasive species, *Erigeron canadensis* L., in China with a maximum entropy model. Glob. Ecol. Conserv..

[39] Muscarella R., Galante P.J., Soley-Guardia M., Boria R.A., Kass J.M., Uriarte M., Anderson R.P. (2014). ENMeval: An R package for conducting spatially independent evaluations and estimating optimal model complexity for MAXENT ecological niche models. Methods Ecol. Evol..

[40] Heinz M., Prospero S. (2025). A modeling approach to determine substitutive tree species for sweet chestnut in stands affected by ink disease. J. For. Res..

[41] Yang L., Li H.E. (2023). Projecting the potential distribution and analyzing the bioclimatic factors of four *Rhododendron* subsect. *Tsutsusi* species under climate warming. J. For. Res..

[42] Lai J.X., Fan M.L., Liu Y., Huang P., Gaisberger H., Li C.H., Zheng Y.Q., Lin F.R. (2025). Habitat suitability modeling of a nearly extinct rosewood species (*Dalbergia odorifera*) under current, and future climate conditions. J. For. Res..

[43] Kaky E., Nolan V., Alatawi A., Gilbert F. (2020). A comparison between Ensemble and MaxEnt species distribution modelling approaches for conservation: A case study with Egyptian medicinal plants. Ecol. Inform..

[44] Cetin I.Z., Ozel H.B., Varol T., Canturk U., Sevik H. (2025). Climate change impacts on *Taxus baccata* distribution and conservation. J. For. Res..

[45] Shi X.M., Zhao J.S., Wang Y.L., Wu G.C., Hou Y.J., Yu C.Y. (2025). Optimized MaxEnt Modeling of *Catalpa bungei* Habitat for Sustainable Management Under Climate Change in China. Forests.

[46] Liu C.R., Berry P.M., Dawson T.P., Pearson R.G. (2005). Selecting thresholds of occurrence in the prediction of species distributions. Ecography.

[47] Yang H., Wei X.T., Zhang M.Y., Zhang J.X. (2025). Potential Distribution and Response to Climate Change in *Puccinellia tenuiflora* in China Projected Using Optimized MaxEnt Model. Biology.

[48] Ma T., Yong D., Xu D.P., He Z.P., Zhuo Z.H. (2026). Potential Distribution Pattern and Ecological Suitability Analysis of Hippophae Tibetana Schltdl in China Based on the MaxEnt Model. Ecol. Evol..

[49] Tian Y., Song J., Cheng B.C., Wei R.B., Zeng Y., Zhang J.K., Zhang J.G., Wang Z.S. (2025). Modeling the Impact of Climate Change on the Distribution of *Populus adenopoda* in China Using the MaxEnt Model. Forests.

